# Comparison of continuous subcutaneous hydromorphone hydrochloride and morphine hydrochloride injection on skin disorders incidence: a retrospective study

**DOI:** 10.1186/s40780-024-00401-6

**Published:** 2024-12-19

**Authors:** Rei Tanaka, Takahiro Hashizume, Tadashi Hisanaga, Shinya Masuda, Junya Sato, Hiroshi Ishikawa, Hironori Tanaka, Akiyoshi Saitoh, Tetsumi Sato, Takeshi Kamoshida, Tetsu Sato, Michihiro Shino

**Affiliations:** 1 Department of Pharmacy, Shizuoka Cancer Center, Shizuoka, Japan; 2 Faculty of Pharmaceutical Sciences, Shonan University of Medical Sciences, Kanagawa, Japan; 3 Faculty of Pharmaceutical Sciences, Tokyo University of Science, Chiba, Japan; 4 Wakakusa Dispensing Pharmacy, Shizuoka, Japan; 5 Division of Palliative Medicine, Shizuoka Cancer Center, Shizuoka, Japan

**Keywords:** Hydromorphone, Morphine, Opioid, Subcutaneous injection, Skin disorders

## Abstract

**Background:**

Continuous subcutaneous administration of injectable opioids is simple and effective; however, skin disorders may occur when high opioid dosages are used. Therefore, we investigated opioid injection drugs with a low risk of skin disorders.

**Methods:**

A retrospective study was conducted using the electronic medical records of patients prescribed 1% hydromorphone hydrochloride or 4% morphine hydrochloride with instructions for continuous subcutaneous administration at Shizuoka Cancer Center from January 2017 to December 2021. The primary endpoint was skin disorders incidence, and the two groups were compared using Cox proportional hazards model analyses and Fisher’s exact test at 5% significance level. Patient background factors expected to influence skin disorders were also investigated, and multivariate logistic analysis of skin disorders incidence was performed.

**Results:**

The incidence of skin disorders in the hydromorphone hydrochloride and morphine hydrochloride groups were 3.7% (1/27 patients) and 28.1% (9/32 patients), respectively, showing a significant difference in two statistical analyses between the two groups (Cox proportional hazards model analyses HR: 0.09, 95% CI: 0.01–0.70, *P* = 0.022. Fisher’s exact test OR: 0.10, 95% CI: 0.01–0.84, *P* = 0.016). In the multivariate analysis, the administration of hydromorphone hydrochloride (OR: 0.04, 95% CI: 0.003–0.48, *P* = 0.012) was also found to have a significant negative correlation with the occurrence of skin disorders. On the contrary, administration period ≥ 28 days (OR: 18.16, 95% CI: 2.22–148.60, *P* = 0.007) was a factor with a significant positive correlation.

**Conclusions:**

Subcutaneous 1% hydromorphone hydrochloride administration had a lower risk of skin disorders than 4% morphine hydrochloride injection. Moreover, prolonging the administration period increased the risk of developing skin disorders. This suggests that a 1% hydromorphone hydrochloride Injection is a good clinical decision for patients who are likely to have a longer administration period and require a higher dosage of injectable opioids.

**Trial registration:**

Retrospectively registered.

## Background

In pharmacotherapy for cancer pain, opioids are key drugs, and the WHO recommends that analgesics be administered “by mouth,” “by the clock,” “for the individual,” and “with attention to detail.” [1] Oral administration should usually be used to minimize patient burden, but injectable administration is used when oral administration itself is difficult, for example, owing to the deterioration of swallowing function or the general condition. The advantages of injectable administration include the ability to maintain stable blood levels through continuous administration and the rapid onset of effects in the rescue administration of opioids. However, injectable drug administration is highly invasive. Intravenous administration is associated with a high risk of vascular injury and bleeding. Therefore, continuous subcutaneous administration of injectable agents is considered preferable for patients with weakened blood vessels or who unconsciously self-remove the injection route owing to various conditions such as delirium [2]. While subcutaneous administration has a low risk of vascular injury, adverse events such as skin redness and induration may occur [3]. 

Strong opioid injections for cancer pain in Japan include 1% morphine hydrochloride, 4% morphine hydrochloride, 1% oxycodone hydrochloride, 0.005% fentanyl citrate, 0.2% hydromorphone, and 1% hydromorphone, all of which can be administered subcutaneously [4–7]. These are examples of how opioids should be used: fentanyl is recommended when renal function is impaired, and oxycodone and fentanyl should be avoided when concomitant CYP3A4 inhibitors or inducers are being used [8]. If continuous subcutaneous injection of multiple opioids is an option after taking those considerations into account, the drug with the lowest incidence of skin disorders should be administered.

Another important aspect of subcutaneous administration of opioid injectables is the upper limit of absorption compared with that of intravenous administration [9]. For example, if the subcutaneous injection dosage of 1% morphine hydrochloride exceeds 1.0 mL/h (< 0.5 mL/h is desirable), 4% morphine hydrochloride should be injected [10–12]. The two options were 1% hydromorphone hydrochloride and 4% morphine hydrochloride injections in patients requiring high subcutaneous opioid dosages (Table 1) [8, 13–18]. 

**Table 1:** List of strong opioid injectable drugs available in Japan

Drug name	Osmotic pressure (approximately)	pH	Daily dosage when continuously administered at 0.1 mL/h without dilution (Dosage converted to morphine hydrochloride injection)
4% Morphine Hydrochloride [13]	0.6	2.5–5.0	96 mg/day (96 mg/day)
1% Morphine Hydrochloride [14]	0.2	2.5–5.0	24 mg/day (24 mg/day)
1% Oxycodone Hydrochloride [15]	1.0	4.5–5.5	24 mg/day (24 mg/day)
0.005% Fentanyl citrate [16, 17]	0.01 1	4.5–6.5 3.9–5.9	0.12 mg/day (6 mg/day)
0.2% Hydromorphone hydrochloride [18]	1.0	3.5–4.5	4.8 mg/day (38.4 mg/day)
1% Hydromorphone hydrochloride [18]	1.0	3.5–4.5	24 mg/day (192 mg/day)

The conversion ratio of each opioid injection is morphine: oxycodone: fentanyl: hydromorphone = 200: 200: 4: 25 from the Guidelines for the Pharmacotherapy of Cancer Pain [8]

**Table 2:** List of patient’s background factors and comparison of the two groups

	Hydromorphone hydrochloride group (*n* = 27)	Morphine hydrochloride group (*n* = 32)	*P*
Age (< 75/≥75)	24/3	28/4	0.75^*)^
Sex (Male/ Female)	10/17	19/13	0.12^*)^
Performance status (≤ 3/4)	5/22	4/28	0.72^*)^
Body mass index (< 25/≥25)	23/4	26/6	0.74^*)^
Concomitant steroid use (Yes/No)	15/12	24/8	0.17^*)^
Concomitant non-opioid analgesics use (Yes/No)	9/18	17/15	0.19^*)^
Concomitant anti-histamine use (Yes/No)	7/20	4/28	0.31^*)^
Dilution with saline solution (Yes/No)	4/23	2/30	0.40^*)^
Starting flow rate (< 0.5 mL/h/≥0.5 mL/h)	25/2	32/0	0.20^*)^
Daily dosage (morphine equivalent < 60 mg/day/≥60 mg/day)	3/24	6/26	0.49^*)^
Administration period (< 28 days/≥28 days)	20/7	26/6	0.54^*)^

^*)^ Fisher’s exact test

**Table 3:** Logistic analysis on factors associated with the development of skin disorders

Factors	Occurrence of skin disorders					Univariate logistic analysis			Multivariate logistic analysis		
	Yes (*n* = 10)			No (*n* = 49)		OR	95% CI	*P*	OR	95% CI	*P*
	*n*	%	*n*		%						
Use of hydromorphone (non-use of morphine)	1	10.0	26		53.1	0.10	0.01–0.99	0.034	0.04	0.003–0.48	0.012
Age ≥ 75	2	20.0	7		14.3	2.20	0.36–18.37	0.391	–	–	–
Male	4	40.0	25		51.0	1.04	0.27–4.06	0.953	–	–	–
Performance status 4	9	90.0	40		81.6	1.76	0.19–15.86	0.616	–	–	–
Body mass index ≥ 25	2	20.0	8		16.3	1.28	0.23–7.19	0.778	–	–	–
Concomitant steroid use	7	70.0	32		65.3	1.24	0.28–5.42	0.775	0.30	0.04–2.25	0.244
Concomitant non-opioid analgesics use	5	50.0	21		42.9	1.33	0.39–5.21	0.479	0.93	0.16–5.63	0.943
Concomitant anti-histamine use	1	10.0	10		20.4	0.43	0.05–3.83	0.452	0.51	0.03–7.56	0.623
Dilution with saline solution	1	10.0	5		10.2	0.99	0.10–9.45	0.992	–	–	–
Starting flow rate ≥ 0.5 mL/h	0	0	2		4.1	–	–	–	–	–	–
Daily dosage (morphine equivalent ≥ 60 mg/day)	9	90.0	41		83.7	1.76	0.19–15.86	0.616	–	–	–
Administration period ≥ 28 day	5	50.0	8		16.3	5.13	1.20-21.91	0.028	18.16	2.22–148.60	0.007

Kato et al. reported a case in which switching from continuous subcutaneous administration of 4% morphine hydrochloride (no dilution) to 1% hydromorphone hydrochloride (2.5× dilution) improved skin induration [19]. Considering the formulation concentration, it was expected that 1% hydromorphone hydrochloride injection would result in a lower risk of developing skin disorders than 4% morphine hydrochloride injection. However, this is the first study to observe both drugs from the start of subcutaneous administration and compared their incidence of skin disorders.

## Methods

### Study participants

Patients who received continuous subcutaneous administration of 1% hydromorphone hydrochloride and 4% morphine hydrochloride without any combination other than saline for cancer pain at the Shizuoka Cancer Center between January 1, 2017, and December 31, 2021, were defined as participants. The observation period was defined as the period during which 1% hydromorphone hydrochloride and 4% morphine hydrochloride were administered. Patients who received intravenous opioid injections and those who had skin disorders prior to the start of continuous subcutaneous opioid injections were excluded from the study.

### Investigation items

The primary endpoint was defined as the cumulative incidence of all skin disorders (induration, redness, bleeding spots, burning, and itching) at Gr 1 or higher using Common Terminology Criteria for Adverse Events Ver 5.0 during the entire study period. Age, sex, Eastern Cooperative Oncology Group Performance Status (PS), Body Mass Index (BMI), concomitant medications, starting flow rate, daily dosage (morphine hydrochloride injection equivalent) at the start of treatment, dilution with saline solution, and administration period were investigated retrospectively from the electronic medical record as background factors related to skin disorders. Concomitant medications were defined as steroids (dexamethasone, betamethasone, prednisolone, methylprednisolone, fludroxycortide, and fluticasone propionate), non-opioid analgesics (loxoprofen, diclofenac, naproxen, sulpyrine, flurbiprofen axetil, and acetaminophen), and anti-histamines (famotidine, ranitidine, diphenhydramine, fexofenadine, and bilastine) administered at least once for approximately three days from the start to end.

### Statistical analyses

The participants were categorized into hydromorphone hydrochloride and morphine hydrochloride injection groups, and the cumulative skin disorders incidence was compared using Cox proportional hazards model analyses and Fisher’s exact test. In addition, age (< 75 / ≥75 years) [20], sex, PS (≤ 3/ 4), BMI (< 25 / ≥25) [21], concomitant medications, starting flow rate (< 0.5 / ≥0.5 mL/h) [10], daily dosage (< 60 / ≥60 mg/day of morphine hydrochloride injection) [22], dilution with saline solution, and administration period (< 28 / ≥28 days) [18] were compared by Fisher’s exact test. Univariate logistic regression analysis was performed to analyze factors influencing skin disorders, such as hydromorphone hydrochloride injection use (or absence of morphine hydrochloride injection), age, sex, general condition, BMI, concomitant medications, starting flow rate, daily dosage, dilution with saline solution, and administration period between the skin disorders occurrence and non-occurrence groups. In addition, background factors with *P* < 0.2 were adopted in the multivariate logistic regression analyses [23]. 

Since concomitant medications have been shown to be a risk factor for subcutaneous injection-derived skin disorders in previous reports [24–28], concomitant steroid use, non-opioid analgesics use, and anti-histamine use were included as factors in the multivariate analysis, even if the *P* value in the univariate analysis was ≥ 0.2. Chi-squared analyses were conducted for the adopted background factors based on the degrees of freedom and scale ratio to test the significance of the regression analysis. All statistical tests were run in Bell Curve for Excel (Social Survey Research Information Co., Ltd.) at 5% statistical significance level.

### Ethical consideration

This study was conducted in compliance with the “Ethical Guidance for a Study in Medicine-Targeted Humans” and was approved by the Institutional Review Board of the Shizuoka Cancer Center (approval number: J2021-174-2023-10-3).

**Fig. 1:**
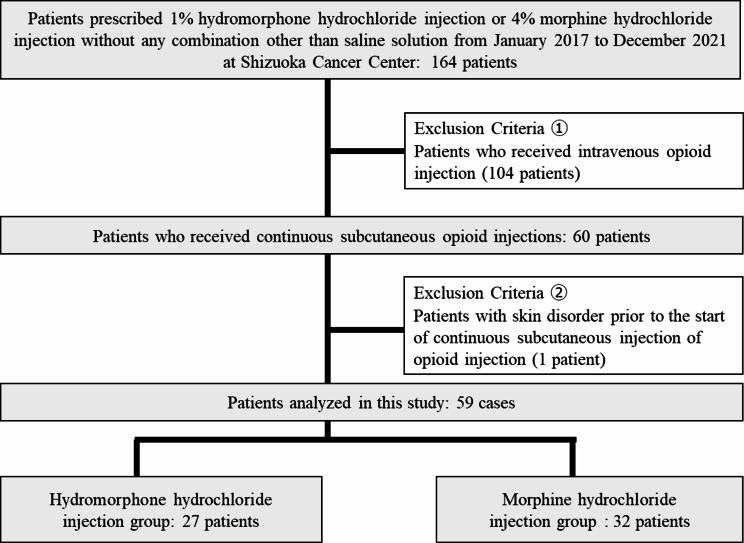
Flow diagram of patient selection. The number of patients excluded in this study are shown

**Fig. 2:**
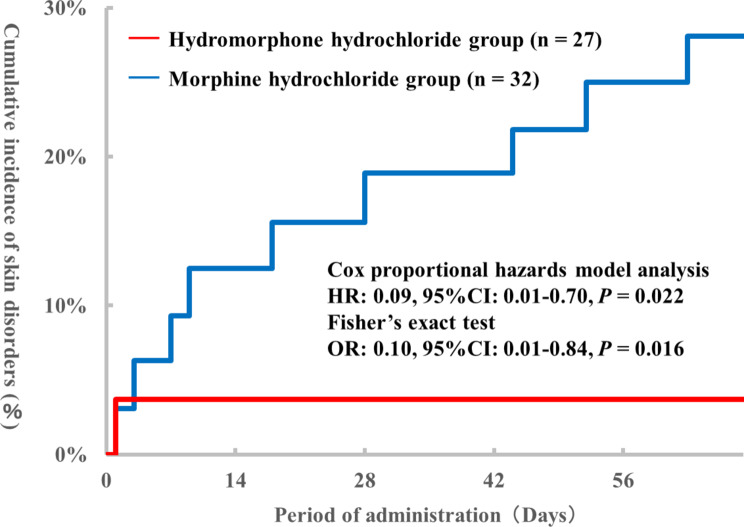
Comparison of cumulative incidence of skin disorders. The date of onset and incidence of skin disorders are shown in hydromorphone hydrochloride group and morphine hydrochloride group

## Results

### Comparative of cumulative incidence of skin disorders

A total of 164 patients were included in this study. Among these, 104 patients receiving intravenous opioids and one patient developing skin disorders prior to starting subcutaneous opioid injections were excluded. Out of the 59 patients included, 27 and 32 were assigned to the hydromorphone hydrochloride and morphine hydrochloride groups, respectively (Fig. 1). The incidence of skin disorders in the hydromorphone hydrochloride and morphine hydrochloride groups were 3.7% (1/27 patients) and 28.1% (9/32 patients), respectively, showing a significant difference in two statistical analyses between the two groups (Cox proportional hazards model analyses HR: 0.09, 95% CI: 0.01–0.70, *P* = 0.022. Fisher’s exact test OR: 0.10, 95% CI: 0.01–0.84, *P* = 0.016). In addition, the cumulative skin disorders incidence in the morphine hydrochloride injection group tended to increase over time (Fig. 2). When patients’ background factors were compared, there were no significant differences between the two groups based on any of the factors (Table 2).

### Logistic analysis of skin disorders occurrence and non-occurrence groups

In univariate logistic regression analysis of the skin disorders occurrence and non-occurrence groups, the background factors with *P* < 0.2 were “use of hydromorphone hydrochloride injection (non-use of morphine hydrochloride injection)” and “administration period (28 ≥ days)” (Table 3). Multivariate logistic regression analysis including these factors and concomitant medications factors showed a significant negative correlation between “use of hydromorphone hydrochloride injection (non-use of morphine hydrochloride injection)” and skin disorders occurrence (OR: 0.04, CI: 0.003–0.48, *P* = 0.012) as well as a significant positive correlation with " administration period ≥ 28 days” (OR: 18.16, CI: 2.22–148.60, *P* = 0.007). We also confirmed that the significance of regression analysis in the present logistic analysis was maintained (*P* = 0.006).

## Discussion

These results suggest that subcutaneous hydromorphone hydrochloride injection carries a lower skin disorders risk than morphine hydrochloride injection. The relationship between the number of days and cumulative incidence shown in Fig. 2 and the results of the multivariate logistics analysis shown in Table 3 suggest that prolonged administration period increases the risk of developing skin disorders.

The factors important for skin tissue damage during subcutaneous injection include the overall formulation osmolality, pH, and concentration of the drug main ingredient of the. Araki et al. reported a higher incidence of erythema and induration during subcutaneous 4% morphine hydrochloride injection than during 1% morphine hydrochloride (10.6% vs. 23.5%) [24]. The morphine hydrochloride concentration was cited as the reason for this result. Table 1 shows that the osmotic pressure of the 1% formulation was closer to that of saline; however, 4% formulation injection had a high incidence of skin disorders, suggesting that the concentration of the main ingredient of the drug significantly affects skin disorders incidence. In the same report, 0.005% fentanyl citrate injection had an even lower incidence of redness and induration. The pharmacological activity of fentanyl citrate is high and the fact that it can be administered at a lower concentration (amount of substance) than morphine hydrochloride preparations may also contribute to the lower incidence of skin tissue damage. The slightly acidic pH (2.5–5.0) of morphine hydrochloride injection should also cause the higher incidence of skin disorders. Kato et al. reported a case in which opioid switching from continuous subcutaneous 4% morphine hydrochloride injection (no dilution) to 1% hydromorphone hydrochloride injection (2.5-fold dilution) improved skin induration [19]. They attributed that this improvement was because the 1% hydromorphone hydrochloride injection had a lower main ingredient concentration than the 4% morphine hydrochloride injection, and the osmolality was closer to that of saline. The results of this study suggest that a similar mechanism may be responsible for the lower cumulative skin disorders incidence with hydromorphone hydrochloride injections than that with morphine hydrochloride injections.

One of the limitations of this study was that opioid selection was left to the discretion of the attending physician, which may have introduced bias. Second, researchers reviewed and mutually audited medical records for the relevant period; however, data on background factors associated with skin disorders may have been missing. As third limitation of this study, the fact that it was a retrospective study might have introduced bias, since the skin detection and evaluation of skin disorders depends on the knowledge and skills of the physicians, nurses, and pharmacists. In addition, the in-dwelling needle must be replaced periodically during continuous subcutaneous injection [10], and the timing of this replacement was not standardized, which may have affected the results. Finally, we performed multivariate analysis of the types of concomitant medications (steroids, non-opioid analgesics, and antihistamines) and found no significant correlation with skin disorders. However, we cannot rule out an influence on the dosage and administration period of concomitant medication. Future prospective studies are needed to address this bias.

The risk factors for skin disorders during continuous subcutaneous injection include age and BMI, which affect the amount of fat in the skin tissue and blood vessel fragility; however, no clear cut-off values for increased risk have been reported for any of these factors. Therefore, in this study, we set the cut-off values at 75 years of age, which is the definition of late elderly in Japan, and at a BMI of 25, which is the standard weight in Japan [20, 21]. In addition, we used 28 days, the maximum duration of subcutaneous hydromorphone hydrochloride injection in prospective clinical trials in Japan, as the standard [18]. The standard flow rate was defined as less than 0.5 mL/h, which is recommended for continuous subcutaneous opioid injections [10]. The cut-off dosage was set at 60 mg/day for morphine injection, which is described as a relatively high dosage by the Ministry of Health guidance on the proper use of ethical drugs [22]. However, because opioid flow rates and dosages fluctuate during administration, the comparable values in this study were limited to the starting point. These cut-off values derived from actual clinical practice were not different according to patient background factors between the two groups (Table 2). And multivariate logistic analysis showed that subcutaneous 1% hydromorphone hydrochloride administration had a lower risk of skin disorders than 4% morphine hydrochloride injection. In addition, it was shown that prolonging the administration period ≥ 28 days increased the risk of developing skin disorders (Table 3).

## Conclusion

The results of this study suggest that hydromorphone hydrochloride is a useful drug with a lower risk of skin disorders than morphine hydrochloride when administered subcutaneously.

## Data Availability

The dataset supporting the conclusions of this article is included within the article.

## Abbreviations

BMI: body mass index
CI: confidence interval
HR: hazard ratio
OR: odds ratio
PS: performance status

## References

[1] World Health Organization. WHO Guidelines for the pharmacological and radiotherapeutic management of cancer pain in adults and adolescents. 2019. https://www.who.int/publications/i/item/9789241550390 30776210

[2] Rietjens JA, van Zuylen L, van Veluw H, van der Wijk L, van der Heide A, van der Rijt CC. Palliative sedation in a specialized unit for acute palliative care in a cancer hospital: comparing patients dying with and without palliative sedation. J Pain Symptom Manage. 2008;36:228–34. 10.1016/j.jpainsymman.2007.10.014:0.1016. Epub 2008 Apr 14. 18411017 10.1016/j.jpainsymman.2007.10.014

[3] Bruno VG. Hypodermoclysis: a literature review to assist in clinical practice. Einstein (Sao Paulo). 2015;13:122–8. 10.1590/S1679-45082015RW2572. Epub 2015 Mar 24. 25807246 10.1590/S1679-45082015RW2572 PMC4946820

[4] Bruera E, Velasco-Leiva A, Spachynski K, Fainsinger R, Miller MJ, MacEachern T. Use of the Edmonton Injector for parenteral opioid management of cancer pain: a study of 100 consecutive patients. J Pain Symptom Manage. 1993;8:525–8. 10.1016/0885-3924(93)90081-6. 7525782 10.1016/0885-3924(93)90081-6

[5] Moulin DE, Kreeft JH, Murray-Parsons N, Bouquillon AI. Comparison of continuous subcutaneous and intravenous hydromorphone infusions for management of cancer pain. Lancet. 1991;337:465–8. 10.1016/0140-6736(91)93401-t. 1704089 10.1016/0140-6736(91)93401-t

[6] Kinnunen M, Piirainen P, Kokki H, Lammi P, Kokki M. Updated clinical pharmacokinetics and pharmacodynamics of oxycodone. Clin Pharmacokinet. 2019;58:705–25. 10.1007/s40262-018-00731-3. 30652261 10.1007/s40262-018-00731-3

[7] Watanabe S, Pereira J, Hanson J, Bruera E. Fentanyl by continuous subcutaneous infusion for the management of cancer pain: a retrospective study. J Pain Symptom Manage. 1998;16:323–6. 10.1016/s0885-3924(98)00095-5. 9846027 10.1016/s0885-3924(98)00095-5

[8] Palliative Medicine Guidelines General Committee, Japanese Society for Palliative Medicine. Clinical guidelines for pharmacological treatment of cancer pain. 2020 version. Tokyo: Kanehara & Co., Ltd.; 2020. (in Japanese).

[9] Palliative Medicine Guidelines General Committee, Japanese Society for Palliative Medicine. Guidelines for infusion therapy of terminally ill cancer patients. 2013 version. Tokyo: Kanehara & Co., Ltd.; 2013. (in Japanese).

[10] Hisanaga T, Yabuki R. Shojo kanwa no tame no dekiru tsukaeru hikatoyo. Tokyo: Nanzando & Co., Ltd.; 2020. (in Japanese).

[11] Adams F, Cruz L, Deachman MJ, Zamora E. Focal subdermal toxicity with subcutaneous opioid infusion in patients with cancer pain. J Pain Symptom Manage. 1989;4:31–3. 10.1016/0885-3924(89)90061-4. 2467954 10.1016/0885-3924(89)90061-4

[12] Kato Y, Kusumi F, Obayashi H. A retrospective study of the incidence of subcutaneous induration induced by hydromorphone and haloperidol. Palliat Care Res. 2020;15:129–34. 10.2512/jspm.15.129. (in Japanese).

[13] The package insert of ANPEC Injection 200 mg in Japan. Sumitomo Pharmaceutical Co., Ltd. https://www.info.pmda.go.jp/go/pack/8114401A3026_1_12/?view=frame&style=XML&lang=ja. Accessed 25 Aug 2024.

[14] The package insert of ANPEC Injection 50 mg 10 mg in Japan. Sumitomo Pharmaceutical Co., Ltd. https://www.info.pmda.go.jp/go/pack/8114401A1082_1_13/?view=frame&style=XML&lang=ja. Accessed 25 Aug 2024.

[15] The package insert of OXIFAST Injection 50 mg 10 mg in Japan. Shionogi Pharmaceutical Co., Ltd. https://www.info.pmda.go.jp/go/pack/8119400A1025_2_05/?view=frame&style=XML&lang=ja. Accessed 25 Aug 2024.

[16] The package insert of Fentanyl Injection. 0.1 mg 0.25 mg in Japan. Daiichi Sankyo Propharma Co., Ltd. https://www.info.pmda.go.jp/go/pack/8219400A1063_1_12/?view=frame&style=XML&lang=ja. Accessed 25 Aug 2024.

[17] The package insert of Fentanyl Injection. 0.1 mg 0.25 mg 0.5 mg in Japan. Terumo Corporation. https://www.info.pmda.go.jp/go/pack/8219400A1071_1_03/?view=frame&style=XML&lang=ja. Accessed 25 Aug 2024.

[18] The package insert of NARUVEIN Injection 2 mg 10 mg in Japan. Daiichi-Sankyo Propharma Co., Ltd. https://www.info.pmda.go.jp/go/pack/8119401A1020_1_07/?view=frame&style=XML&lang=ja. Accessed 25 Aug 2024.

[19] Kato Y. Kusumi FEffective switching from high concentration morphine citrate to high concentration hydromorphone citrate for subcutaneous induration and uncontrollable pain: a case report. Palliat Care Res. 2019;14:39–42. 10.2512/jspm.14.39. (in Japanese).

[20] Ouchi Y, Rakugi H, Arai H, Akishita M, Ito H, Toba K, et al. Redefining the elderly as aged 75 years and older: proposal from the Joint Committee of Japan Gerontological Society and the Japan Geriatrics Society. Geriatr Gerontol Int. 2017;17:1045–7. 10.1111/ggi.13118. Epub 2017 Jul 2. 28670849 10.1111/ggi.13118

[21] World Health Organization. Prevalence of overweight among adults, BMI ≥ 25. https://www.who.int/data/gho/indicator-metadata-registry/imr-details/2390

[22] Ministry of Health, Labour and Welfare. Guidance on the appropriate use of medical narcotics – guidance on the use and management of medical narcotics in the treatment of cancer pain and chronic pain; 2017 revision. (in Japanese). https://www.niph.go.jp/h-crisis/archives/90733

[23] Peduzzi P, Concato J, Kemper E, Holford TR, Feinstein AR. A simulation study of the number of events per variable in logistic regression analysis. J Clin Epidemiol. 1996;49:1373–9. 10.1016/s0895-4356(96)00236-3. 8970487 10.1016/s0895-4356(96)00236-3

[24] Araki K, Haraguchi M. Both switching from a winged needle to a small plastic intravenous catheter and adding dexamethasone to continuous subcutaneous infusion (CSCI) successfully treated inflammatory skin changes caused by CSCI. Palliat Care Res. 2012;7:112–20. 10.2512/jspm.7.112. (in Japanese).

[25] Reymond L, Charles MA, Bowman J, Treston P. The effect of dexamethasone on the longevity of syringe driver subcutaneous sites in palliative care patients. Med J Aust. 2003;178:486–9. 10.5694/j.1326-5377.2003.tb05321.x. 12741933 10.5694/j.1326-5377.2003.tb05321.x

[26] Fagien S, McChesney P, Subramanian M, Jones DH. Prevention and Management of Injection-related adverse effects in Facial aesthetics: considerations for ATX-101 (deoxycholic acid injection) treatment. Dermatol Surg. 2016;42:S300–4. 10.1097/dss.0000000000000898. 27787270 10.1097/DSS.0000000000000898

[27] Wu CC, Chen WJ, Cheng JJ, Hsieh YY, Lien WP. Local dermal hypersensitivity from dobutamine hydrochloride (dobutrex solution) injection. Chest. 1991;99:1547–8. 10.1378/chest.99.6.1547. 1828022 10.1378/chest.99.6.1547

[28] Imakado S, Hamada K, Atsumi Y. Subcutaneous antihistamine injection is effective to control a local allergic reaction to human insulin. Diabetes Res Clin Pract. 2009;85:e42–3. 10.1016/j.diabres.2009.05.013. 19520450 10.1016/j.diabres.2009.05.013

